# Association between Interlukin-6 (IL-6), Interlukin-10 (IL-10) and depression in patients undergoing Hematopoietic stem cell transplantation

**Published:** 2015-04-01

**Authors:** Maria Tavakoli-Ardakani, Maryam Mehrpooya, Mahshid Mehdizadeh, Abbas Hajifathali, Alireza Abdolahi

**Affiliations:** 1 Department of Clinical Pharmacy, School of pharmacy and Pharmaceutical Sciences Research Center, Shahid Beheshti University of Medical Sciences, Tehran, Iran; 2 School of Pharmacy, Hamedan University of Medical Sciences, Hamedan, Iran; 3 Taleghani Bone Marrow Transplantation Center, Taleghani Hospital and Pediatric Congenital Hematologic Disorders Research Center, Shahid Beheshti University of Medical Sciences ,Tehran, Iran; 4 Taleghani Bone Marrow Transplantation Center, Taleghani Hospital, Shahid Beheshti University of Medical Sciences, Tehran, Iran; 5 Vali-Asr Hospital, Tehran University of Medical Sciences, Tehran, Iran

**Keywords:** Depression, Hematopoietic stem cell transplantation, hs-CRP, Inflammatory cytokines, IL-6

## Abstract

**Background:** The release of pro-inflammatory cytokines is responsible for the variety of behavioral, neuro-endocrine and neuro-chemical alterations in psychiatric condition. In this study we evaluate relation between depression and IL-6 and IL-10 in patients undergoing hematopoietic stem cell transplantation (HSCT).

**Materials and Methods:** 66 patients in this cross-sectional study from July 2013 until August 2014 for HSCT interred the study and were assessed for depression using Hospital Anxiety and Depression Scale (HADS). Serum interleukin (IL)-6, (IL)-10 and high sensitive C-reactive protein (hs-CRP) were assessed on the same time. Association between these biomarkers with depression was evaluated using SPSS version 20.

**Results:** A total of 66 patients with the mean age of 41.18+13.92 and 41.95+12.35 years old in non depressed and depressed group respectively were enrolled in this study. Patients with depression showed significantly higher levels of serum IL-6 and the IL-6-to-IL-10 ratio compared to patients without depression (p<0.001).There was no statistically significant association between IL-10 and hs-CRP with depression in this group of the patients.

**Conclusions:** High IL-6 level has significant association with depression in patients undergoing HSCT. In conclusion, since IL-6 can affect the outcomes after HSCT and depression was associated with increased serum IL-6 level, early identification of depression can be beneficial in these patients.

## Introduction

 Hematopoietic stem cell transplantation (HSCT) is an important medical procedure in the treatment of several malignant, solid tumor and some severe nonmalignant diseases.^1^ Approximately 40% of patients after HSCT develop serious post-transplant complications.^2^ Psychological distress has been observed in one-third of complicated HSCT patients and may be more severe in patients undergoing bone marrow transplantion.^3^^-^^5^ Depression can influence the patient’s outcome after HSCT, via various direct and indirect mechanisms. Depressed HSCT patients have poor compliance to their therapy and have poor nutrition, sleep disorder, poor exercise, and also a higher risk for substance abuse and smoking.^6^^,^^7^ Psychosocial factors can affects on outcomes after HSCT via catecholamines, glucocorticoids, inflammation, angiogenesis and cellular immune function and infectious susceptibility that cause complication after transplantation such as acute graft-versus-host disease (aGVHD) and hepatic veno-occlusive disease (VOD) and less efficient response to injury or infection.^8^

 Psychological distress has influence on the quality of life as well as survival in cancer patients.^9^ Psychosocial factors also may have an effect on survival in HSCT patients through an immunologic mechanism. Recently, anxiety and depression have been associated with slower immune recovery as assessed by white blood cell (WBC) count following transplantion.^10^^-^^12^ Also, the adverse effects of depression has been identified as a risk factor for early post-transplant mortality.^13^

 In recent years, the role of inflammatory markers has been raised in the chronic course of depression.^14^ In several studies, the increased level of inflammatory markers, including CRP, TNF-α, IL-1, IL-6 and IL-2R, in depressed subjects as compared with non-depressed subjects was observed.^15^^-^^17^ A cytokine with anti-inflammatory effects such as IL-10 are also linked to acute immune challenge and may be implicated in depression.^18^^,^^19^ Thus, several studies showed that inflammatory cytokines can increase the risk of transplant-related mortality (TRM). Studies have shown that high peak levels of hs-CRP as an acute phase protein were associated with the occurrence of major transplant-related complications (MTC).^20^ In several studies, the adverse relationship between the IL-6 levels and survival and also complications after transplantation such as aGVHD was observed.^21^^-^^23^ Also, low capacity of IL-10 production by blood mononuclear cells may contribute to mechanisms involved in the event of MTC.^24^

 Considering to importance of cytokines in psychological distress, we investigated the association of depression with pro-inflammatory markers of IL-6, and hs-CRP and anti-inflammatory cytokine (IL-10) in patients undergoing either autologous or allogeneic transplant at Taleghani BMT transplantation center affiliated to Shahid Beheshti University of Medical Sciences, in Tehran, Iran.

## MATERIALS AND METHODS

**The study population and design of the study**

 In this cross sectional study, all the eligible patients who were admitted for either autologous or allogeneic HSCT from July 2013 until August 2014 were included in our study. Patients with cognitive dysfunction, active infection, acute medical or any other surgical condition and those with inflammatory disorders such as autoimmune diseases, history consumption of anti-depressant, anti-anxiety or anti-inflammatory agents within one month before the study were excluded. All the patients signed their consent form. The study was approved by the Ethics Committee of Shahid Beheshti University of Medical Sciences in Tehran, Iran. Diagnosis of depression was defined by a psychiatric and the severity of depression was evaluated via Hospital Anxiety and Depression Scale (*HADS*) questionnaire. 

 All patients were informed how to complete the questionnaire by the same researcher. The study population was divided into two subgroups. The first group consisted of depressed patients and the second group included patients without depression. 

**Assessment of depression symptoms**

 The HADS questionnaire was used to assess the patients’ depression based on self-reports. This questionnaire contains a 14-item scale of which seven items measure the anxiety and the other seven measure the depression and each item is composed the scales of ‘0’ to ‘3’. The possible total HADS score ranges from 0 (no depression, no anxiety) to 21 (maximal depression or anxiety). Then all scores of the items are added to create a total score for each patient. The HADS survey has been validated to measure psychological distress among Iranian patients and have high sensitivity and specificity for the diagnosis of depression and anxiety among the population.^25^ A HADS score with cut off score ≥8 was defined for differentiating individuals with anxious from non-anxious and depressed from non-depressed patients.

**Measurement of inflammatory cytokines**

 Blood samples were drawn on admission from all patients who were included in our study. Samples were collected and centrifuged for 10 min. within a few hours of sampling. Serum was removed and frozen at −70°C until completion of the samples. High-sensitivity sandwich enzyme-linked immunosorbent (ELISA) kits (Immulite, DPC Cirrus Inc., Los Angeles, CA, USA; Amersham Bioscience, USA; Amersham Bioscience, USA; Bender Med Systems, Austria, respectively) were used for the determination of hs-CRP and cytokines (IL-6, IL-10) concentrations.

**Statistical analyses**

 Data was analyzed using SPSS for windows (SPSS Inc., Chicago, IL, USA) version 20. For normally distributed continuous variables mean ± SD and for non-normally distributed continuous median (interquartile range) were used for analysis. Number or percentages was used for nominal variables. Normality of the variables distributions was assessed by Kolmogorov-Smirnov test. The distribution of categorical variables was compared between two groups using chi-square and Fisher exact tests. 

 Independent sample t-test and Mann–Whitney U-test were used to compare normally and non-normally distributed variables respectively between the two groups of the study. The correlation between depression scores with continues variables were tested by Pearson's correlation test. Binary logistic regression was used to measure the effect of significant variables that had been tested by univariate analyses with depression. P value less than 0.05 was considered as significant. 

## Results

 A total of 88 patients were enrolled in our study. Of these, 22 patients were excluded due to active infection (4 patients), severe psychiatric disorders (3 patients) and consumption of anti-inflammatory medications (4 patients), consumption of anti-depressant medications (5 patients) and non-eligibility to participate in the study (6 patients). Therefore, a total of 66 patients (28 subjects in non-depressed group and 38 subjects in depressed group) were enrolled in this study. Medical and demographic characteristics of the patients are given in Table 1. As seen in Table 1, significant difference in gender between two groups was observed (p value=0.01) and other variables did not show significant differences between two groups. Twenty eight of these 66 patients (42.4%) had HADS score less than 8 which defined as non-depressed group. The rest of the patients had HADS score equal or more than 8 which defined as depressed group. The median HADS scores of patients with and without depression were median: 11 [IQR: 8 to16], p = <0.001) vs. median: 3 [IQR: 0 to 6], p = <0.001), respectively.

**Table1:** Medical and demographic characteristics of the HSCT patients in two groups (N = 66)

	**Non-depressed group** **(n=28)**	**Depressed ** **group** **(n=38)**	**P value**
**Age (years), mean** **+** ** standard**	41.18+13.926	41.95+12.357	0.81
**Sex (female)**	8 (28.5%)	22 (59%)	0.01
**Married**	24 (85.7%)	33 (86.8%)	0.89
**BMI, median (IQR)**	26.08 (18-34)	26.19 (18-34)	0.91
**Education** **Below high school** **High school** **Above high school**	8 (28.6%) 14 (50%) 6 (21.4%)	12 (31.6) 16 (42%) 10 (26.3%)	0.80
**Type of transplantation** **Autologous** **Allogeneic**	22(78.6%) 6(21.4%)	24 (63.2%) 14 (36.8%)	0.17
**Diagnosis of diseases leading to transplantation** **AML** **ALL** **MM** **NHL** **HL** **Other**	4 (14.3%) 2 (7.1%) 10 (35.7%) 14 (14.3) 7 (25%) 1 (3.6%)	7 (18.4%) 7 (18.4%) 16 (42.1%) 2 (5.3%) 5 (13.2%) 1 (2.6%)	0.50
**Co-morbidity** **Diabetes mellitus** **Hypertension** **Ischemic heart disease** **Fatty liver** **Hypothyroidism**	4 14.3%) 5 (17.9%) 0 (0%) 3 (10.7%) 2 (7.1%)	6 (15.8%) 5 (13.2%) 3 (7.9%) 5 (13.2%) 1 (2.6%)	0.86 0.59 0.25 1.00 0.57

**HADS:** Hospital Anxiety and Depression Scale, **ALL:** acute lymphocytic leukemia, **AML:** acute myelogenous leukemia, **HL:** Hodgkin's lymphoma, **NHL:** Non-Hodgkin's lymphoma, **MM:** Multiple myeloma, **BMI:** Body mass index

 Among continuous variables, patients with depression had significantly higher concentrations of IL-6 and IL-6 to IL-10 ratios (P<0.001). Serum levels of IL-10 and hs-CRP did not differ significantly between two groups (Table 2). 

**Table 2:** Comparison of inflammatory and anti-inflammatory markers between two groups

	**Non-depressed ** **group** **(n=28)**	**Depressed ** **group** **(n=38)**	**P ** **value**
**IL-6 ** **(pg/mL)**	58.50 (4.5-112.0)	101.05 (45.0-640.0)	<0.001
**IL-10 ** **(pg/mL)**	88 (55-316)	82.60 (26-640)	0.25
**hs-CRP ** **(mg/L)**	3 (1-75)	3 (1-75)	0.93
**IL-6 / IL-10**	0.51 (0.30-1.15)	1.21 (0.83-2.34)	<0.001

Data has been presented as median (IQR), hs-CRP: high-sensitivity C-reactive protein, IL-10: interleukin-10; IL-6: interleukin-6, IQR: Interquartile range

 Serum levels of the mentioned cytokines were also compared in patients with different diagnoses leading to transplantation (Table 3). Serum levels of the mentioned cytokines were also compared in patients with different type of transplantation as shown in Table 4. Analysis of correlations between HADS scores within inflammatory, anti-inflammatory markers and some variables associated with depression showed that HADS score were significantly correlated with serum concentrations of IL-6 and IL-6 / IL-10 (P<0.001) (Table 5). There was not a statistically significant correlation between IL-10, hs-CRP and other variables with HADS score. The multivariate logistic regression test after adjusting for body mass index, age, sex, education level, marital status showed there is a direct effect of IL-6 with depression (P<0.001, odds ratio [OR] = 1.05; 95% confidence interval [CI] = 1.02–1.09) (Table 6).

## Discussion

 This study indicates a significant correlation between serum IL-6 level and depression in patients undergoing HSCT. We observed that depression was significantly associated with increased serum IL-6 level in the HSCT patients. The prevalence of depression was 57.6%. In several studies, the prevalence of psychiatric disorders varied from 14% to 41%, depending on the study design, types of evaluation and time of evaluation of these problems at different times of the patients' disease. ^26^^-^^28^ The incidence of depression was somehow high in our study group compatible with another Iranian study in HSCT patients.^29^

 We did not find any significant correlation between depression in terms of age, education level, marital status, BMI, different diagnosis of disease leading to transplant, co-morbidity, serum level of hs-CRP and anti-inflammatory cytokine IL-10. However, there was significant correlation between depression and sex. Since depression was more common in female than male population^2^ it was predictable result.

 Our result showed there was significant correlation between depression and serum IL-6 level. This result is similar to several other studies that have been done in depressed patients. Studies showed that there is a bidirectional relationship between depression and inflammation, so that depression can cause up-regulation of inflammatory mediators and thereafter the inflammatory cytokines can cause depression behaviours.^30^ Recent studies have indicated that increased serum pro-inflammatory cytokines such as IL-6, IL-1, TNF-α, IL-2, IFN-α and other inflammatory cytokine are associated with depression.^15^^,^^31^^,^^32^

 Consistent with the role of IL-6 in depression, the results of the study by Hannestad et al. showed that anti-depressant agents such as SSRIs can reduce serum level of inflammatory cytokine, IL-6 and TNF-α.^33^ Several studies have showed that increased level of IL-6 can affect the occurrence of complications such as infection, aGVHD, VOD, and rate of hematological recovery.^34^^,^^35^ So, depression can also affect the outcome and survival after transplantation through the increased serum level of pro-inflammatory cytokine such as IL-6.

 Our study found no significant correlation between depression and serum hs-CRP level. In several studies, the serum level of hs-CRP had increased in depressed patients.^15^^,^^16^ In a recent meta-analysis on longitudinal studies increased hs-CRP level was associated with increased risk of subsequent depression.^36^ Ford et al, and Ma et al. showed that the association between depression and the increased serum hs-CRP level was much stronger in female gender than male.^37^^,^^38^ Contrary to previous results, we found no significant correlation between depression and serum hs-CRP level. This might be related to the small sample size in our study compared with other studies and paucity of male patients in depressed group compared to non-depressed group.

**Table 3:** Comparing of biomarkers with different diagnosis leading to transplantation in HSCT patients

		**AML** **(n=11)**	**ALL** **(n=9)**	**MM** **(n=26)**	**NHL** **(n=6)**	**HL** **(n=12)**	**P value**
**IL-6 (pg/mL)**		95.10 (55.10-140.90)	89.90 (61.90-690)	91.25 (4.5-640)	68.10 (33-152.9)	72.70 (32-530)	0.25
**IL-10 (pg/mL)**	86.20 (68.50-640)		75.90 (26-133.9)	87.75 (41.40-588)	93.86 (55-112)	86.05 (58.20-215)	0.25
**hs-CRP (mg/L)**		3 (1-14)	3 (1-6)	3 (1-71)	7 (3-75)	3 (3-75)	0.07
**IL-6 / IL-10**		1.01 (0.12-1.87)	1.28 (0.73-4.78)	1.04 (0.01 -8.59)	0.84 (0.29-1.60)	0.76 (0.22 -5.22)	0.21

Data has been presented as median (IQR), **ALL:** acute lymphocytic leukemia, **AML: **acute myelogenous leukemia, **HL: **Hodgkin's lymphoma, **NHL: **Non-Hodgkin's lymphoma, **MM: **Multiple myeloma, **hs-CRP: **high-sensitivity C-reactive protein, **IL-10: **interleukin-10; **IL-6: **interleukin-6

**Table 4:** Comparing serum levels of markers in HSCT patients with different type of transplantation

	**Allogeneic** **(n=20)**	**Autologous** **(n=46)**	**P** **value**
**IL-6 (pg/mL)**	89.6 (72.2-11.3)	81.1(49-106.6)	0.23
**IL-10 (pg/mL)**	78.5(68.7-105.9)	88(76.7-142.5)	0.09
**hs-CRP (mg/L)**	3(3-4)	3.5(3-11.5)	0.10
**IL-6 / IL-10**	1.2(0.8-1.5)	0.8(3-1.4)	0.18

**hs-CRP:** high-sensitivity C-reactive protein, **IL-10:** interleukin-10, **IL-6:** interleukin-6

 We did not also find any significant difference in serum IL-10 level between two groups. Different results were reported about role of IL-10 in depression in various studies.^27^ In the study by Dhabhar et al. the results showed lower serum IL-10 level in depressed patients as compared to non-depressed patients.^39^ While Simon et al found increases in serum IL-10 level in depressed patients.^40^ Our results was similar to Huang et al and O'Brian et al studies which showed no differences in the serum IL-10 level in depressed patients as compared to non-depressed patients.^41^^,^^42^

 For the evaluation of the balance between pro- and anti-inflammatory markers, we assessed the serum concentrations of IL-6 to IL-10 ratio. Our results showed that there is a relationship between increased IL-6-to-IL-10 ratios with depression in patients undergoing HSCT (P<0.001).

**Table5:** Variables correlation with depression score according to HADS

**Factors**	**Depression score** **Correlation **	**p-value**
**Age**	0.05	0.66
**BMI**	0.04	0.69
**hs-CRP**	-0.09	0.46
**IL-6**	0.49	<0.001
**IL-10**	-0.13	0.29
**IL-6 / IL-10**	0.37	<0.001

**hs-CRP:** high-sensitivity C-reactive protein, **IL-10:** interleukin-10, **IL-6:** interleukin-6, **HADS:** Hospital Anxiety and Depression Scale, **BMI:** Body mass index

**Table 6:** Results of logistic regression analysis for correlation of IL-6 with depression

	**B** a	**Standard** **error**	**Odds ratio**	**95% CI**	**P value**
**Age**	0.06	0.04	1.06	.986-1.155	0.10
**Sex**	-2.18	0.88	0.11	.020-.644	0.01
**BMI**	0.15	0.11	1.16	.934-1.461	0.17
**Education**	0.66	0.53	0.19	.674-5.572	0.21
**Marrital Status**	1.11	0. 72	3.07	.492-17.772	0.12
**Type of transplantation**	0.88	0.89	2.42	.424-13.873	0.32
**IL-6**	0.05	0.01	1.05	1.023-1.092	<0.001

*p <0.05, BMI: Body mass index, IL-6: interleukin-6

a Coefficient for the constant,

 Several studies showed a association between pro- and anti-inflammatory cytokines dysregulation with depression symptoms.^43^^,^^44^ Similar to our study, in Dhabhar et al. study showed that depressed subjects expressed significantly higher IL-6/IL-10 ratios.^39^ Also, Taraz et al. have shown a significant increase in the IL-6/IL-10 ratio in depressed hemodyalysis patients.^45^

## CONCLUSION

 Inflammatory markers have been evaluated in several studies on general depressed patients and we found only a few studies according to inflammatory markers and depression relationship in HSCT patients.

 Our study showed potentially important contribution of increased level of IL-6 and IL-6/IL-10 ratio in depressed patients undergoing HSCT. According to the negative effects of IL-6 in post-transplantation outcomes and increased level of this cytokine in depressed patients, it is needed to further investigate the effect of inflammatory and anti-inflammatory cytokines on outcomes of HSCT depressed patients as well as evaluate the effects of the treatment of depression on post-transplantation outcome and the mentioned mediators.
